# Assessing Quantitative Resistance against *Leptosphaeria maculans* (Phoma Stem Canker) in *Brassica napus* (Oilseed Rape) in Young Plants

**DOI:** 10.1371/journal.pone.0084924

**Published:** 2014-01-15

**Authors:** Yong-Ju Huang, Aiming Qi, Graham J. King, Bruce D. L. Fitt

**Affiliations:** 1 School of Life and Medical Sciences, University of Hertfordshire, Hatfield, Hertfordshire, United Kingdom; 2 Department of Plant Pathology and Microbiology, Rothamsted Research, Harpenden, Hertfordshire, United Kingdom; 3 Southern Cross Plant Science, Southern Cross University, Lismore, Australia; Zhejiang University, China

## Abstract

Quantitative resistance against *Leptosphaeria maculans* in *Brassica napus* is difficult to assess in young plants due to the long period of symptomless growth of the pathogen from the appearance of leaf lesions to the appearance of canker symptoms on the stem. By using doubled haploid (DH) lines A30 (susceptible) and C119 (with quantitative resistance), quantitative resistance against *L. maculans* was assessed in young plants in controlled environments at two stages: stage 1, growth of the pathogen along leaf veins/petioles towards the stem by leaf lamina inoculation; stage 2, growth in stem tissues to produce stem canker symptoms by leaf petiole inoculation. Two types of inoculum (ascospores; conidia) and three assessment methods (extent of visible necrosis; symptomless pathogen growth visualised using the GFP reporter gene; amount of pathogen DNA quantified by PCR) were used. In stage 1 assessments, significant differences were observed between lines A30 and C119 in area of leaf lesions, distance grown along veins/petioles assessed by visible necrosis or by viewing GFP and amount of *L. maculans* DNA in leaf petioles. In stage 2 assessments, significant differences were observed between lines A30 and C119 in severity of stem canker and amount of *L. maculans* DNA in stem tissues. GFP-labelled *L. maculans* spread more quickly from the stem cortex to the stem pith in A30 than in C119. Stem canker symptoms were produced more rapidly by using ascospore inoculum than by using conidial inoculum. These results suggest that quantitative resistance against *L. maculans* in *B. napus* can be assessed in young plants in controlled conditions. Development of methods to phenotype quantitative resistance against plant pathogens in young plants in controlled environments will help identification of stable quantitative resistance for control of crop diseases.

## Introduction

With growing concern about world-wide food shortages and climate change [1], protecting food crops against pathogens that cause epidemic diseases is more important than ever. Using resistance against pathogens in crop cultivars is one of the most economical and environmentally friendly methods for control of crop diseases. Two types of cultivar resistance used are quantitative resistance and qualitative resistance [1]–[4]. Quantitative resistance, which is usually controlled by several genes, is often a ‘partial’ resistance that does not prevent pathogens from colonisation of plants but decreases symptom severity and/or epidemic progress over time [5]–[7]. By contrast, qualitative resistance is usually controlled by single, dominant resistance (*R*) genes and often effective in preventing pathogens from colonisation of plants [8]–[12]. Whilst *R* gene-mediated qualitative resistance is ‘complete’ resistance, which follows a gene-for-gene interaction, it is generally less durable than quantitative resistance because pathogen populations often rapidly evolve for virulence against the *R* genes [13]–[16].

Many arable crops rely on quantitative partial resistance that has been selected by breeding ‘field’ resistance into modern cultivars for control of their diseases. This reliance on resistance is essential for small-holder farmers in many regions of the world where use of fungicides is prohibitively expensive. In regions where crops are grown intensively on larger farms, the reliance on resistance for disease control will increase if use of certain effective fungicides is not permitted by legislation. However, it has been difficult to breed cultivars with quantitative resistance because of the difficulty in assessing it. By contrast, *R* gene-mediated qualitative resistance can be selected reliably by phenotyping in young plants (with distinct resistant and susceptible phenotypes after inoculation). Traditionally, selection for quantitative resistance has relied on field assessments of disease severity made towards the end of the cropping season before harvest [17]–[19]. Thus, quantitative resistance has also been referred to as ‘adult plant resistance’ [3] because it has been difficult to assess it in young plants. Furthermore, due to the influence of genotype by environment interactions [16], [19], identification of stable quantitative resistance has required large replicated field plot experiments at different locations in different years, which is very costly and time-consuming. There is a need to develop new methods to select for quantitative resistance in arable crops that are rapid, cheap and reliable.

Phoma stem canker, caused by *Leptosphaeria maculans*, is an economically important disease on oilseed rape (*Brassica napus*) in Europe, North America and Australia, causing world-wide losses worth more than £1000 M (at a price of £300 t^−1^) per cropping season, despite use of fungicides [20]–[21]. In Europe, these losses will increase if use of the most effective fungicides is no longer permitted by EU legislation (EU directive 91/414, http://ec.europa.eu/food/plant/protection/index_en.htm). Epidemics of phoma stem canker are initiated by air-borne *L. maculans* ascospores (sexual spores) [18], [22], which produce germ tubes that infect the leaves of winter oilseed rape in autumn (October/November), causing leaf lesions [23]–[24]. Conidia (asexual spores), produced in pycnidia (asexual fruiting bodies) on leaf lesions and spread by rain-splash, can cause secondary leaf infections [25]. From leaf lesions, *L. maculans* grows symptomlessly along the leaf vein/petiole to reach the stem where, in spring/summer (April-July), it causes stem cankers that result in yield losses [9], [18].

Both quantitative resistance and *R* gene-mediated qualitative resistance against *L. maculans* have been identified in *B. napus* [3], [8], [26]–[27]. *R* gene-mediated resistance against *L. maculans* operates in leaves to prevent leaf lesion development and thus prevent subsequent stem canker development [9], [13]–[14]. Quantitative resistance against *L. maculans* is a partial resistance that does not prevent leaf lesion development but decreases the severity of stem cankers [6], [28].

*R* gene-mediated resistance against *L. maculans* can be selected in young plants by scoring lesion phenotypes after inoculation of cotyledons or leaves [8], [14]. However, *R* gene-mediated resistance against *L. maculans* usually loses its effectiveness within three cropping seasons of widespread use in commercial cultivars because of selection for virulence within the variable *L. maculans* populations. For example, the resistance genes *Rlm1* (introduced into several winter oilseed rape cultivars in France) and *LepR3* (introduced into the Australian cultivar Surpass 400 from *B. rapa* var. s*ylvestris*) were rendered ineffective after 3 years of commercial use [13]–[14], [29]. This is very costly for breeders since introduction of new resistance into elite commercial cultivars can take 10–15 years [20].

By contrast, quantitative resistance is considered to be more durable than *R* gene-mediated resistance against *L. maculans* [3], [30]. However, it has been difficult to select for quantitative resistance against *L. maculans* due to the long period of symptomless growth after initial development of leaf lesions [6]. Furthermore, if effective *R* genes are present, it is difficult to assess quantitative resistance because *R* gene-mediated qualitative resistance operates in leaves to prevent the growth of the pathogen from leaf lesions to stems. Currently, selection of cultivars with quantitative resistance generally relies on winter oilseed rape field experiments in which stem canker severity is assessed just before harvest [18], [30]–[31]. If quantitative resistance can be assessed in young plants, it will not only accelerate the process of breeding for resistance but also save money for the industry. A study of growth of *L. maculans* from the cotyledon to the hypocotyl showed that stem canker symptoms can be produced on hypocotyls after cotyledon inoculation in controlled conditions [28]. However, it was not clear whether resistance to growth of *L. maculans* to cause canker in hypocotyls is the same as that in stems. Recently, a petiole inoculation method has been used to compare pathogenicity of different *L. maculans* isolates and to screen different cultivars in terms of stem canker development at a young-plant stage in controlled conditions [32]–[33]. However, there is a need for further work to develop methods to test for quantitative resistance in young plants.

This paper reports work to investigate new methods for assessing quantitative resistance against *L. maculans* in controlled environment experiments with young plants of two oilseed rape doubled haploid (DH) lines differing in quantitative resistance, using different types of inoculum and different inoculation and assessment methods.

## Materials and Methods

**Figure 1:**
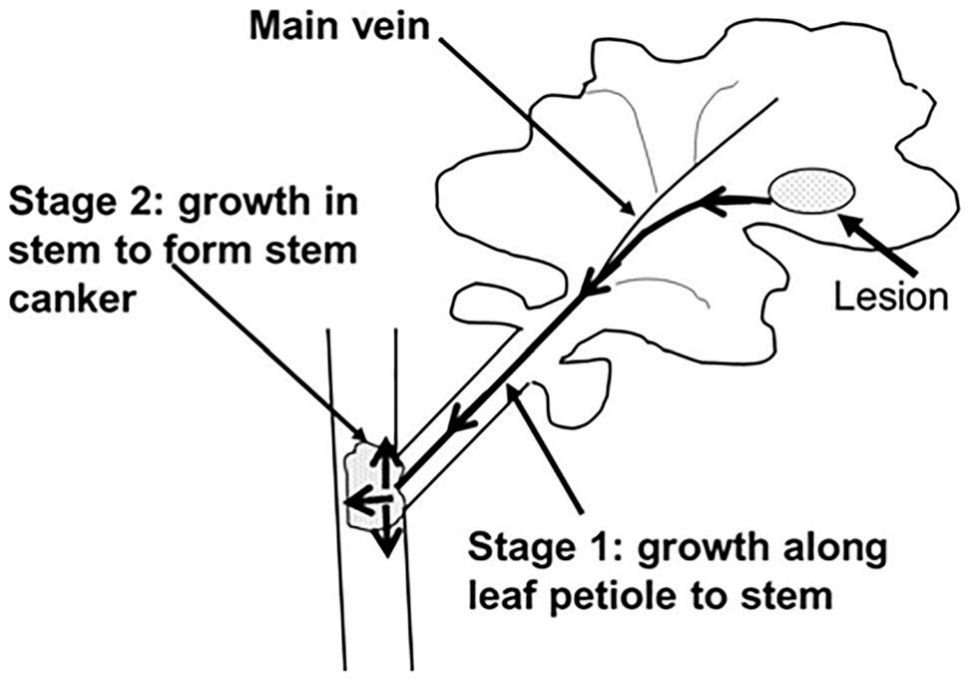
Stages of *Leptosphaeria maculans* growth in oilseed rape. Assessment of quantitative resistance against growth of *L. maculans* in oilseed rape was considered in two stages; stage 1, resistance against growth from phoma leaf lesion along the main leaf vein and petiole to the stem; stage 2, resistance against growth in stem to produce stem canker; both stages were investigated in controlled environment experiments.

In this work, assessment of quantitative resistance against growth of *L. maculans* in oilseed rape was considered in two stages; stage 1, resistance against growth along leaf veins/petioles towards the stem; stage 2, resistance against growth in stem tissues to produce stem canker symptoms (Fig. 1). Inoculation methods, type of *L. maculans* inoculum, number of plants inoculated, design of experiments and assessment methods used in each of the controlled environment experiments are described in Table 1.

**Table 1:** Inoculation methods, type of *Leptosphaeria maculans* inoculum, number of plants inoculated, design of experiments and assessment methods used in each of the controlled environment experiments with the doubled haploid (DH) lines A30 or C119.

Exp.	Inoculum^1^	Design	No. of plants inoculated^2^	Assessment method
*L. maculans* growth in leaf, leaf lamina inoculation				
LExpt 1	Conidia (GFP)	Randomised block	8×2 plants, 3 leaves per plant	Lesion area^3^, distance grown (DG) along vein/petiole viewed by GFP
LExpt 2	Conidia (GFP)	Complete randomised	8 plants, 3 leaves per plant	Lesion area, DG viewed by GFP
LExpt 3	Ascospores	Randomised block	5×2 plants, 3 leaves per plant	DG assessed by extent of necrosis, *L. maculans* DNA in leaf petiole (qPCR)
LExpt 4	Ascospores	Complete randomised	12 plants, 3 leaves per plant	DG assessed by extent of necrosis, qPCR
LExpt 5	Ascospores	Complete randomised	8 plants, 2 leaves per plant	Lesion area, DG assessed by extent of necrosis, qPCR
*L. maculans* growth in stem, leaf petiole inoculation				
PExpt 1	Conidia (GFP)	Complete randomised	10 plants, 2 petioles per plant	Stem canker score, extent of *L. maculans* growth in stem viewed by GFP
PExpt 2	Ascospores	Complete randomised	5 plants, 3 petioles per plant	Stem canker score, *L. maculans* DNA in stem (qPCR)
PExpt 3	Ascospores	Complete randomised	12 plants, 2 petioles per plant	Stem canker score, qPCR
PExpt 4	Ascospores	Complete randomised	12 plants, 2 petioles per plant	Stem canker score, qPCR

^1^ *L. maculans* ascospores obtained from naturally infected oilseed rape stem base debris collected in August 2007; conidia were produced by GFP-transformed isolate ME24/3.13.

**Figure 2:**
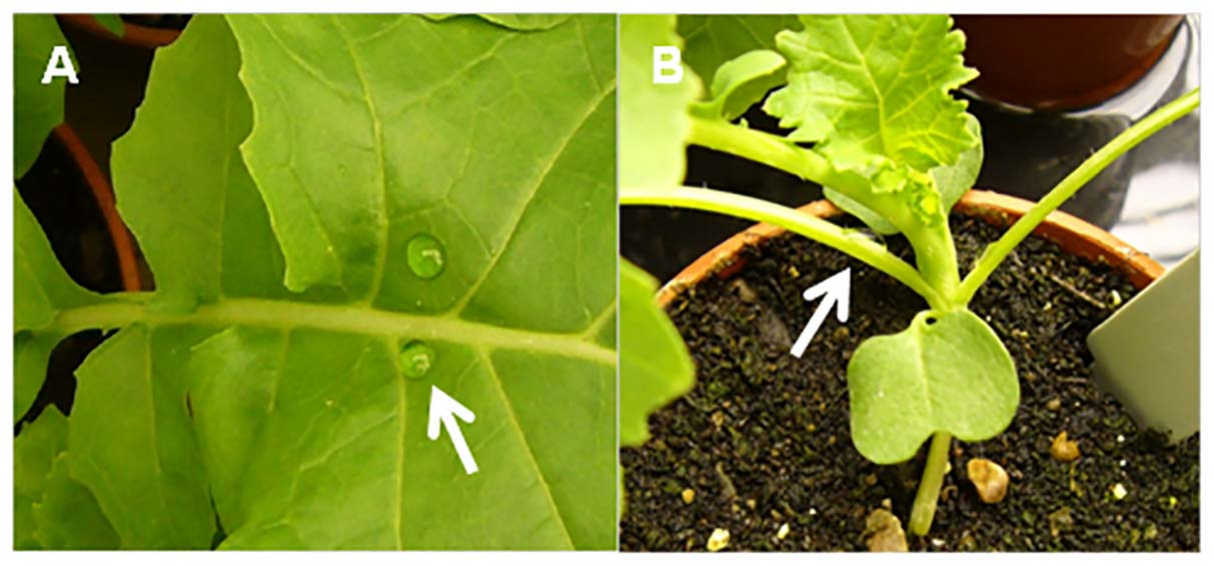
Leaf lamina and leaf petiole inoculation. Inoculation of oilseed rape leaf lamina (A) or leaf petiole (B) with conidia or ascospores of *Leptosphaeria maculans*. The arrows indicate the droplets of spore suspension (either conidia or ascospores) placed on the lamina (A) or petiole (B) of leaves of doubled haploid lines A30 (susceptible) or C119 (with quantitative resistance). Plants were inoculated at 21 days after sowing when they had three fully expanded leaves.

2 Plants were inoculated when they had three fully expanded leaves. For details of leaf lamina or leaf petiole inoculation, see Fig. 2. All experiments were done at 20°C with alternating 12 h light/12 h darkness.

3 The lesion area was estimated by multiplying the lesion length by lesion width.

### Preparation of plant material and pathogen inoculum

DH lines A30 and C119, selected from the mapping population developed from a cross between winter oilseed rape Darmor-*bzh* (with quantitative resistance against *L. maculans* and *R* gene *Rlm9*) and the spring oilseed rape Yudal (without quantitative resistance or *R* genes) [30], were used for all the experiments. These two DH lines were derived from microspore cultures of a single F_1_ hybrid plant from the cross ‘Darmor-*bzh* × Yudal’. Use of DH lines can decrease potential variation associated with genetic background. Since no effective major resistance gene segregates in this DH population, ‘field’ resistance provides an assessment of quantitative resistance against *L. maculans*. When ‘field’ resistance was assessed by scoring stem canker at the end of the cropping season in winter oilseed rape field experiments in France, A30 developed severe phoma stem cankers whilst C119 developed much less severe stem cankers [27], [30]. Similar results were obtained when these two lines were assessed in field experiments in the UK (the mean disease score on a 0–4 scale was 1.9 for A30 and 0.6 for C119 in 2007/2008, and 2.7 for A30 and 1.3 for C119 in the 2008/2009 cropping season). Results of these field experiments show that A30 is susceptible and does not have quantitative resistance whereas C119 has good quantitative resistance against *L. maculans*. Furthermore, these two lines do not have the dwarfing gene (*bzh*) and do not differ greatly in terms of maturity date and plant height. Molecular markers in QTL regions for resistance to *L. maculans* showed that the difference in resistance between these two DH lines was mainly due to resistance inherited from the resistant parent (Personal communication, Dr Regine Delourme). Therefore, these two DH lines were chosen for this work.

Plants of lines A30 or C119 were grown in pots (9 cm diameter) containing a peat-based compost mixed with a soluble fertiliser (WE Hewitt & Sons Ltd, UK). Plants were initially grown in a glasshouse (20–23°C) and thinned to one plant per pot 10 days after sowing. Three weeks after sowing, when each plant had three expanded leaves, the plants were transferred to a controlled environment cabinet (20°C day/20°C night, 12 h light/12 h darkness, light intensity 210 µmol m^−2^s^−1^) for 24 h before inoculation. After inoculation, plants were kept in the cabinet until the end of the experiments.

Both ascospores and conidia were used as inoculum. Although line C119 has resistance gene *Rlm9* (A30 has no *R* genes), the *L. maculans* populations throughout Europe are 100% virulent against *Rlm9* [8], [34], so ascospores from natural populations were used. Both the *L. maculans* isolates used as conidial inoculum, ME24 (*AvrLm1, avrLm2, avrLm3, avrLm4, avrLm5, AvrLm6*, *AvrLm7, avrLm9*) and GFP-expressing isolate ME24/3.13 (GFP-transformed ME24; all work involving the use and storage of genetically modified *L. maculans* was done under Defra licence no. PHL 174E/5443/01/2007), are virulent against *Rlm9*. Conidial suspensions of isolates ME24 and ME24/3.13 were prepared from 12-day-old cultures on V8 agar [9]. The concentration of conidia was adjusted to 10^7^ conidia mL^−1^ using a haemocytometer slide. Since phoma stem canker epidemics in crops are initiated by ascospores released from stem debris, *L. maculans* ascospores produced under natural conditions were also used as inoculum. An ascospore suspension of *L. maculans* was prepared from naturally infected UK oilseed rape stem base debris of the cultivar Courage collected after harvest in August 2007 (pieces of stem debris with mature pseudothecia were stored at −20°C until required), using the method described by Huang *et al*. [23]. The concentration of ascospores was adjusted to 10^4^ ascospores mL^−1^ using a haemocytometer slide.

### Growth of *L. maculans* in leaf petioles

In natural conditions in Europe, phoma stem cankers are usually initiated from phoma lesions on leaves of winter oilseed rape crops in the previous autumn [18], [22]. Therefore, to investigate the growth of *L. maculans* from leaf lesions towards the stems, experiments were done by inoculation of leaf laminas. Before inoculation, each leaf lamina was rubbed gently using a wet tissue so that the droplet of inoculum would not run off. For inoculation with conidia, the rubbed sites were wounded using a sterile pin and a 10 µL droplet of conidial suspension was placed on each wounded site. For inoculation with ascospores, the rubbed sites were not wounded and a 15 µL droplet of ascospore suspension was placed on each site. Each leaf had two inoculation sites, one on each side of the main vein (Fig. 2A). The first two or three fully expanded leaves of each plant were inoculated.

Two experiments were done with conidia of GFP-expressing *L. maculans* isolate ME24/3.13 and three experiments were done with ascospores (Table 1). After inoculation, plants in trays were covered with tray covers to maintain high relative humidity for 48 h (after inoculation with ascospores) or 72 h (after inoculation with conidia). The time from inoculation to the appearance of phoma leaf lesions was recorded in each experiment. The maximum length and width of each lesion were measured at 17–18 days post inoculation (dpi), then the lesion area (mm^2^) was estimated by multiplying the lesion length by lesion width.

For leaf inoculation with conidia of GFP-expressing *L. maculans*, the distance grown by *L. maculans* from the inoculation site along leaf the vein/petiole towards the stem was assessed by measuring extent of appearance of GFP fluorescence. At 22–25 dpi, the inoculated leaves were detached at the place where the petiole joins the stem (leaf scar). The leaves were viewed using a Leica MZ FLIII stereo-microscope. For observation of GFP fluorescence, filter GFP2 from Leica Microsystems (Milton Keynes, UK) was used.

For leaf inoculation with ascospores, the growth of *L. maculans* along the leaf vein/petiole towards the stem was measured by assessing the extent of visible necrosis and by quantification of the pathogen DNA in the tissue using quantitative PCR (qPCR). At 18–20 dpi, the inoculated leaves were detached at the leaf scar. The distance from the inoculation site on the leaf lamina to the furthest visible necrosis on the leaf petiole was measured on each leaf, then a piece of leaf petiole 8 cm long (measured from the inoculation site) was placed in a 15 mL tube to be freeze-dried for DNA extraction and qPCR.

### Growth of *L. maculans* in stem tissues

To ensure that *L. maculans* reached the stem before the leaf abscission, the petioles of first two or three leaves of each plant were inoculated (Table 1). Before inoculation, the leaf petiole (1 cm from the stem) was gently rubbed using a wet tissue so that the droplet of inoculum would not run off. Then the leaf petiole was wounded using a sterile pin and either a 10 µL droplet of conidial suspension or a 12 µL droplet of ascospore suspension was placed over the wound (Fig. 2B). One experiment was done with conidia of GFP-expressing *L. maculans* isolate ME24/3.13 and three experiments were done with ascospores (Table 1). After inoculation, plants in trays were covered with tray covers to maintain high relative humidity for 48 h (after inoculation with ascospores) or 72 h (after inoculation with conidia). The time from inoculation to the appearance of first phoma stem canker symptoms at the leaf scar on the stem was recorded for each experiment.

For petiole inoculation with conidia of GFP-expressing isolate ME24/3.13, the growth of *L. maculans* in the stem was measured both by assessing stem canker severity and by observation of extent of GFP fluorescence within stem tissues. After inoculation, plants were regularly assessed for appearance of visible canker symptoms at the leaf scars of inoculated leaf petioles. At 87 dpi, the inoculated plants were sampled by cutting their stems at the soil surface and these stems were viewed using a Leica MZ FLIII stereo microscope for appearance of GFP fluorescence at the leaf scar of each inoculated leaf. The stems were then cut horizontally at the leaf scar of the inoculated leaf to assess the internal severity of stem canker on a 0–4 scale; where 0 = healthy; 1 = 1–25% stem cross-section necrotic; 2 = 26–50% stem cross-section necrotic; 3 = 51–75% stem cross-section necrotic; 4 = 76–100% stem cross-section necrotic. The scale was modified from that of Zhou *et al*. [35]. Then stems were cut vertically to assess internal vertical spread of GFP-expressiong *L. maculans* along the stem cortex or pith using a Leica MZ FLIII stereo-microscope.

For leaf petiole inoculation with ascospores, the growth of *L. maculans* in the stem was measured by assessing stem canker severity and by quantification of the pathogen DNA using qPCR. At 46 dpi, stems of the inoculated plants were sampled by cutting the stem at the soil surface. The stems were then cut horizontally at the leaf scar of the inoculated leaf to assess the internal severity of stem canker on the 0–4 scale. After stem canker assessment, a piece of each stem (5 cm long) was placed in a 50 mL tube to be freeze-dried for DNA extraction and qPCR.

### Quantification of *L. maculans* DNA in leaf petioles and stems

Freeze-dried individual leaf petioles or stems were ground into powder using a mortar and pestle. DNA was extracted from each ground individual leaf petiole or stem using a DNA extraction kit (DNAMITE Plant Kit, Microzone Ltd, West Sussex, UK). The amount of *L. maculans* DNA in each leaf petiole or stem was quantified using a SYBR green quantitative PCR. The primers LmacF and LmacR, specific for amplification of the internal transcribed spacer (ITS) region of ribosomal DNA of *L. maculans*, that have been previously used for identification [36] and quantification [6] of *L. maculans* were used. All qPCR reactions were done in duplicate and each reaction volume was 20 µL, including 0.6 µL of primers at a final concentration of 300 nM, 10 µL of SYBR Green JumpStart Tag ReadyMix (Sigma, UK), 0.08 µL of ROX internal reference dye and the 50 ng DNA sample. Nuclease-free water (Sigma, UK) was used as the no-template control. All reactions were done in 96×0.2 mL PCR plates (ABgene) covered with cap strips, using a Stratagene Mx3000P quantitative PCR machine. The thermocycling profile consisted of an initial cycle of 95°C for 2 min, followed by 40 cycles of 95°C for 15 s, 60°C for 30 s, 72°C for 45 s and a read step at 83°C for 15 s, then a dissociation stage (thermal profile: 95°C 1 min, 60°C 1 min, 95°C 15 s). In each qPCR run, a standard dilution series consisting of 10000, 1000, 100, 10 and 1 pg of DNA of *L. maculans* isolate ME24 was included to produce a standard curve. A standard curve was generated by plotting the amount of *L. maculans* DNA against the threshold cycle (*Ct*) value for the series of dilutions. The amount of *L. maculans* DNA for each unknown sample was extrapolated from the *Ct* value and the value obtained from the standard curve using Stratagene MxPro-Mx3000 P v3.2 software.

### Statistical analysis

For experiments to assess growth of *L. maculans* in leaf petioles, the data on leaf lesion area, distance grown by *L. maculans* along leaf petioles and amount of *L. maculans* DNA in leaf petioles were analysed by analysis of variance to assess the differences between DH lines A30 and C119 or between different experiments. For experiments to assess growth of *L. maculans* in stem tissues, the data on stem canker severity and amount of *L. maculans* DNA in stems were analysed by analysis of variance to assess the differences between DH lines A30 and C119 or between different experiments. To compare different methods used to assess quantitative resistance to *L. maculans* at growth stages 1 or 2, linear regressions were done for individual experiments; these regression lines were compared to assess whether they differed in intercept and/or slope for different experiments. The data on amount of *L. maculans* DNA in petioles or stems were log_10_-transformed before regression analysis. For stage 1 assessments, linear regressions were done for leaf lesion area against distance grown or amount of *L. maculans* DNA and for amount of *L. maculans* DNA against distance grown in the leaf vein/petiole. For stage 2 assessments, linear regressions were done for amount of *L. maculans* DNA in the stem against stem canker severity score. All the analyses were done using GENSTAT statistical software [37].

## Results

### Growth of *L. maculans* in leaf petioles

**Figure 3:**
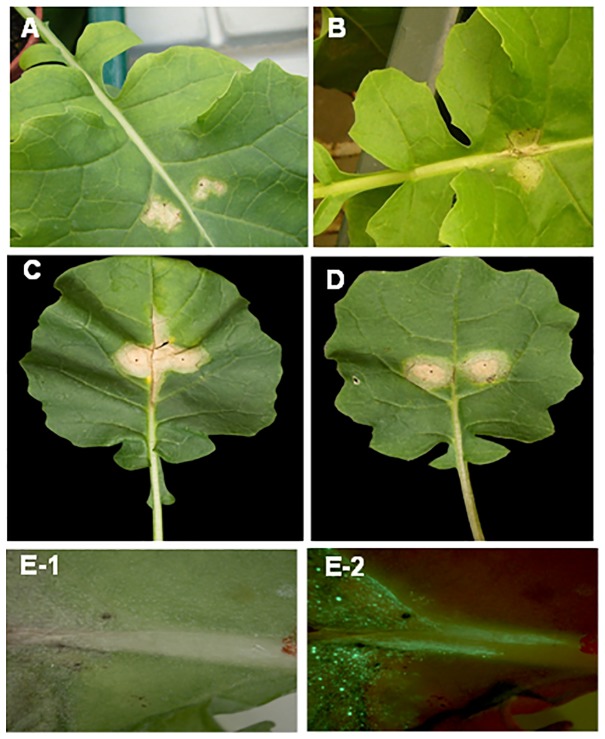
Phoma leaf spot symptoms produced in controlled conditions. Phoma leaf spot symptoms on leaves of doubled haploid lines A30 (susceptible) or C119 (with quantitative resistance) inoculated with conidia or ascospores of *Leptosphaeria maculans*. Lesions on leaves of A30 at 15 days post inoculation (dpi) with conidia (A) or 10 dpi with ascospores (B); lesions on leaves of A30 (C) and C119 (D) at 22 dpi; symptomless growth of GFP-expressing *L. maculans* (isolate ME24/3.13) along a leaf vein towards the petiole of A30 viewed with brightfield illumination (E-1) or a GFP filter (E-2) at 18 dpi.

There were differences between conidial inoculum and ascospore inoculum in incubation period (time from inoculation to appearance of lesions) for phoma leaf lesion development, with the incubation period shorter for ascospores than for conidia. Phoma leaf lesions were observed at 7 dpi for plants inoculated with ascospores (Fig. 3B) but phoma leaf lesions were not observed on plants inoculated with conidia until 10 dpi (Fig. 3A). There was no difference between lines A30 and C119 in incubation period or visual appearance of phoma leaf spot symptoms, whether the plants were inoculated with conidia or ascospores.

**Figure 4:**
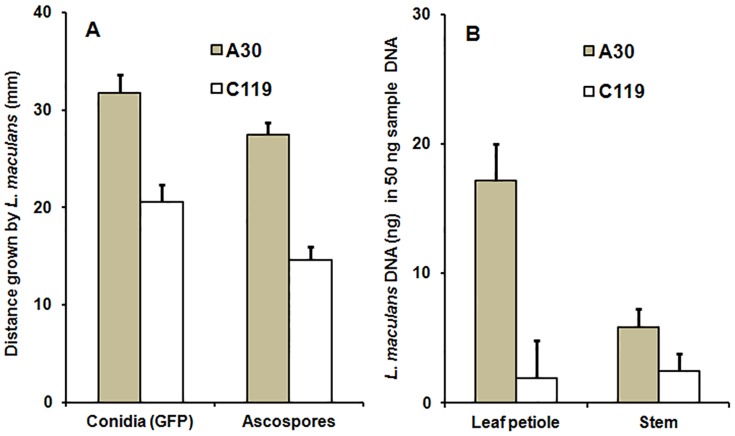
Distance grown along leaf petioles and *Leptosphaeria maculans* DNA in petioles and stems. Leaf laminas or leaf petioles of doubled haploid lines A30 (susceptible) or C119 (with quantitative resistance) were inoculated with conidia of GFP-expressing *L. maculans* isolate ME24/3.13 or ascospores from a natural population. The distance grown by *L. maculans* along the leaf vein/petiole towards the stem (leaf lamina inoculation, Fig. 2A) was measured by assessing extent of GFP fluorescence at 22–25 days post inoculation (dpi) for conidial inoculation (A) or by assessing extent of the visible necrosis (A) and by quantification of amount of *L. maculans* DNA (B) using qPCR at 18–20 dpi for ascospore inoculation. The growth of *L. maculans* in stem tissue (petiole inoculation, Fig. 2B) was assessed by quantification of amount of *L. maculans* DNA using qPCR (B) at 46 dpi inoculation with ascospores. Bars show standard errors (A, 28 d.f. for conidia and 54 d.f. for ascospores; B, 54 d.f. for leaf petiole and 50 d.f. for stem).

In experiments with conidia of GFP-expressing isolate ME24/3/.13 (LExpt 1 & 2; Table 1), the leaf lesions on A30 were larger and the distances grown by *L. maculans* in petioles of A30 were greater than those on C119 (Fig. 3C, d). At 17 dpi, leaf lesion area (average of LExpt 1 & 2) on A30 (60.7 mm^2^) was significantly larger than on C119 (27.4 mm^2^) (*P*<0.001, 28 d.f., SED 7.4). GFP-expressing *L. maculans* (Fig. 3E) was observed in petioles of both A30 and C119; the distance grown by *L. maculans* (average of LExpt 1 & 2) was greater in petioles of A30 than in petioles of C119 (Fig. 4A) (*P*<0.001, 28 d.f., SED 1.6). There was a significant difference between LExpt 1 and LExpt 2 in distance grown by GFP *L. maculans* (*P*<0.05, 28 d.f., SED 1.6), since the distance grown by GFP *L. maculans* was assessed at 22 dpi in experiment LExpt 1 and 25 dpi in LExpt 2. However, there was no significant interaction between experiment and DH line in distance grown along the leaf petiole.

In experiments with ascospore inoculum (LExpt 3, 4 & 5; Table 1), the distance grown (average of LExpt 3, 4 & 5) by *L. maculans* (measured by the extent of necrosis) was greater in petioles of A30 than in petioles of C119 (*P*<0.001, 54 d.f., SED 1.3) (Fig. 4A). There was a significant difference between the three experiments in distance grown by *L. maculans* (*P*<0.001, 54 d.f., SED 1.4). However, there was no significant interaction between experiment and DH line. The difference between these three experiments may have occurred because the distance grown in leaf petioles was assessed at 18 dpi in LExpt 3 and at 20 dpi in LExpt 4 and LExpt 5, because there was no significant difference between LExpt 4 and LExpt 5. When symptomless growth of *L. maculans* in petioles was measured by qPCR, there was a significant difference between A30 and C119 in the amount of *L. maculans* DNA (average of LExpt 3, 4 & 5), with the amount of *L. maculans* DNA greater in leaf petioles of A30 than that in leaf petioles of C119 (*P*<0.001, 54 d.f., SED 2.9; Fig. 4B). There was no difference between experiments in amount of *L. maculans* DNA. Leaf lesion area was assessed in LExpt 5 (not assessed in LExpt 3 and LExpt 4); the leaf lesion area on A30 (327.5 mm^2^) was larger than on C119 (137.6 mm^2^) at 18 dpi (*P*<0.01, 14 d.f., SED 0.6).

**Figure 5:**
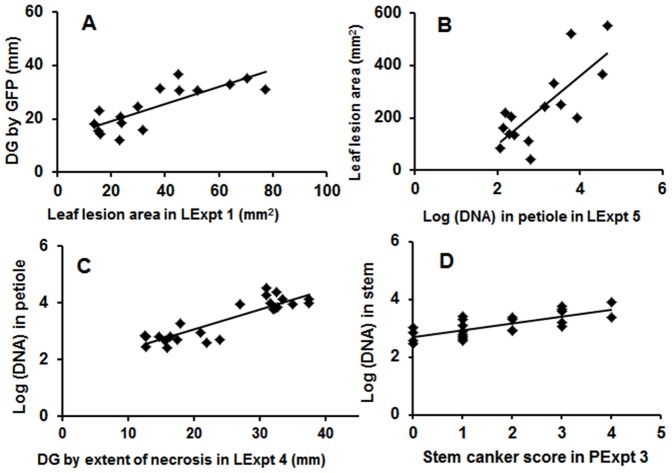
Relationships between different methods for assessment of quantitative resistance. Different methods were used to assess growth of *Leptosphaeria maculans* in leaf petioles or in stems of doubled haploid lines A30 (susceptible) or C119 (with quantitative resistance). Relationships between leaf lesion area and distance grown (DG) by *L. maculans* in the leaf petiole viewed by GFP in experiment LExpt 1 (A; *R* ^2^ = 0.64) or amount of *L. maculans* DNA in leaf petiole in experiment LExpt 5 (B; *R* ^2^ = 0.59); or between amount of *L. maculans* DNA and DG in leaf petiole viewed by extent of necrosis in experiment LExpt 4 (C; *R* ^2^ = 0.78); or between amount of *L. maculans* DNA in the stem and stem canker severity score in experiment PExpt 3 (D; *R* ^2^ = 0.56). The amount of *L. maculans* DNA in the petiole or stem was log_10_-transformed. Details of these experiments are presented in Table 1.

For the experiments with conidial inoculum, there were significant linear relationships between distance grown (*d*) by *L. maculans* and leaf lesion area (*f*) in the leaf petiole in LExpt 1 (*d* = 0.32*f*+12.7, *R* ^2^ = 0.64, *P*<0.001, 14 d.f.) (Fig. 5A) and LExpt 2 (*d* = 0.31*f*+14.1, *R* ^2^ = 0.62, *P*<0.001, 13 d.f.). Comparison of these two regression lines showed that there was no significant difference in intercept (*P*>0.85) or slope (*P*>0.71) between them. A significant linear relationship was observed in experiment LExpt 5 with ascospores (*d* = 3.90*f*+10. 7, *R* ^2^ = 0.40, *P*<0.01, 14 d.f.). There was also a significant linear relationship between leaf lesion area (*f*) and *L. maculans* DNA (*n*) in the leaf petiole (*f* = 130.8*n*−163.0, *R* ^2^ = 0.59, *P*<0.001, 13 d.f.) (Fig. 5B). For the three experiments with ascospore inoculum, there were significant linear relationships between *L. maculans* DNA (*n*) and distance grown (*d*) by *L. maculans* in experiments LExpt 3 (*n* = 0.5*d*+3.0, *R* ^2^ = 0.58, *P*<0.001, 18 d.f.), LExpt 4 (*n* = 0.7*d*+1.6, *R* ^2^ = 0.78, *P*<0.001, 22 d.f.; Fig. 5C) and LExpt 5 (*n* = 0.7*d*+1.7, *R* ^2^ = 0.54, *P*<0.005, 13 d.f.). Comparison of these regression lines showed that the slope did not differ significantly (*P*>0.36) but the intercepts differed significantly (*P*<0.001) between them.

### Growth of *L. maculans* in stem tissues

**Figure 6:**
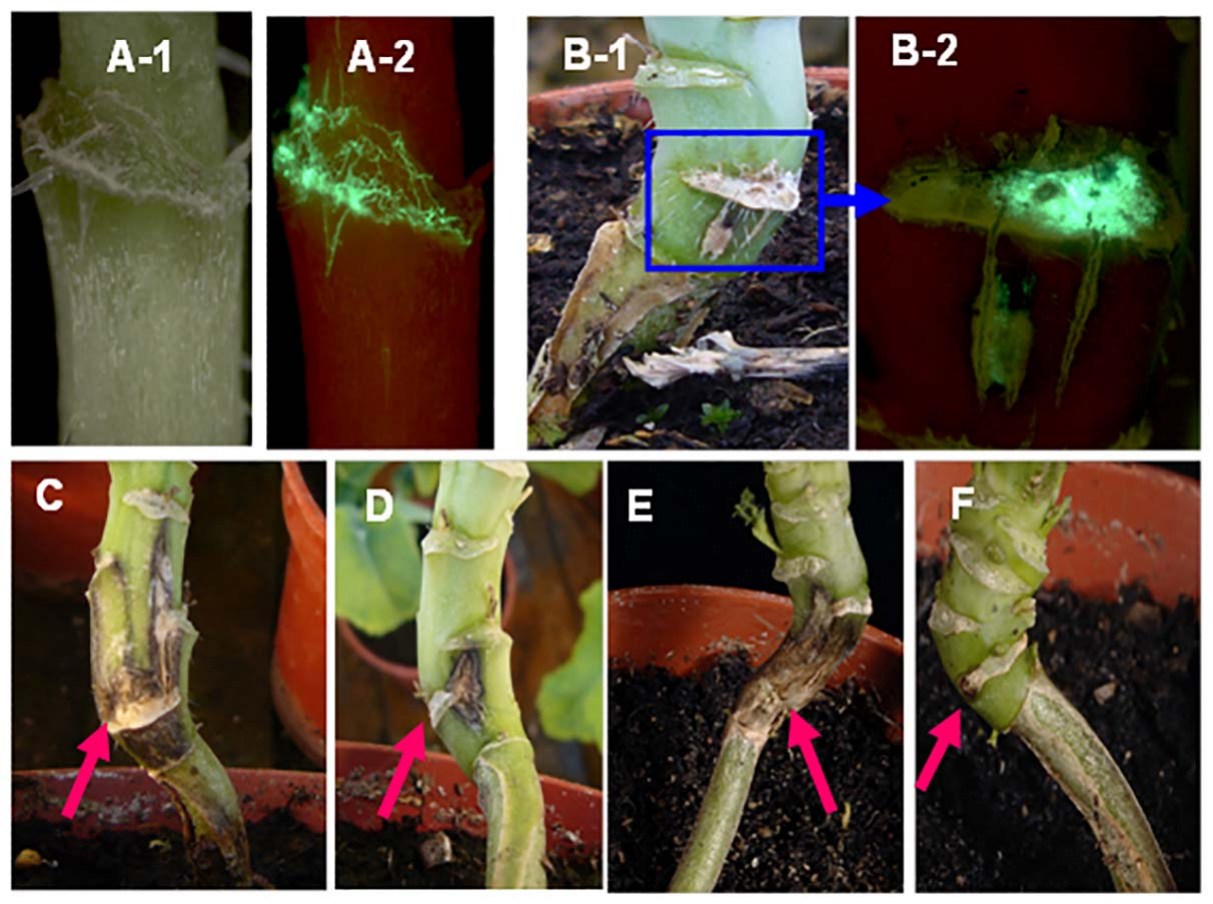
Phoma stem canker symptoms produced in controlled conditions. Leaf petioles of oilseed rape doubled haploid lines A30 (susceptible) or C119 (with quantitative resistance) were inoculated with conidia of GFP-expressing *Leptosphaeria maculans* isolate ME24/3.13 (A, B, C) or ascospores produced on stem debris under natural conditions (E, F). No visible stem canker symptom at the leaf scar (A-1) of A30 after the inoculated leaf had abscised at 25 days post inoculation (dpi) but growth of the pathogen was observed using a GFP2 filter (A-2). Stem canker was visible at the leaf scar of A30 at 36 dpi (B-1) and growth of *L. maculans* was visualised by GFP fluorescence (B-2) (the selected region in B-1 was viewed using a GFP2 filter). Symptoms at leaf scars of A30 (C, E) or C119 (D, F) at 87 dpi with conidia (C, D) or at 31 dpi with ascospores (E, F) (arrows indicate the leaf scars of inoculated leaves).

**Figure 7:**
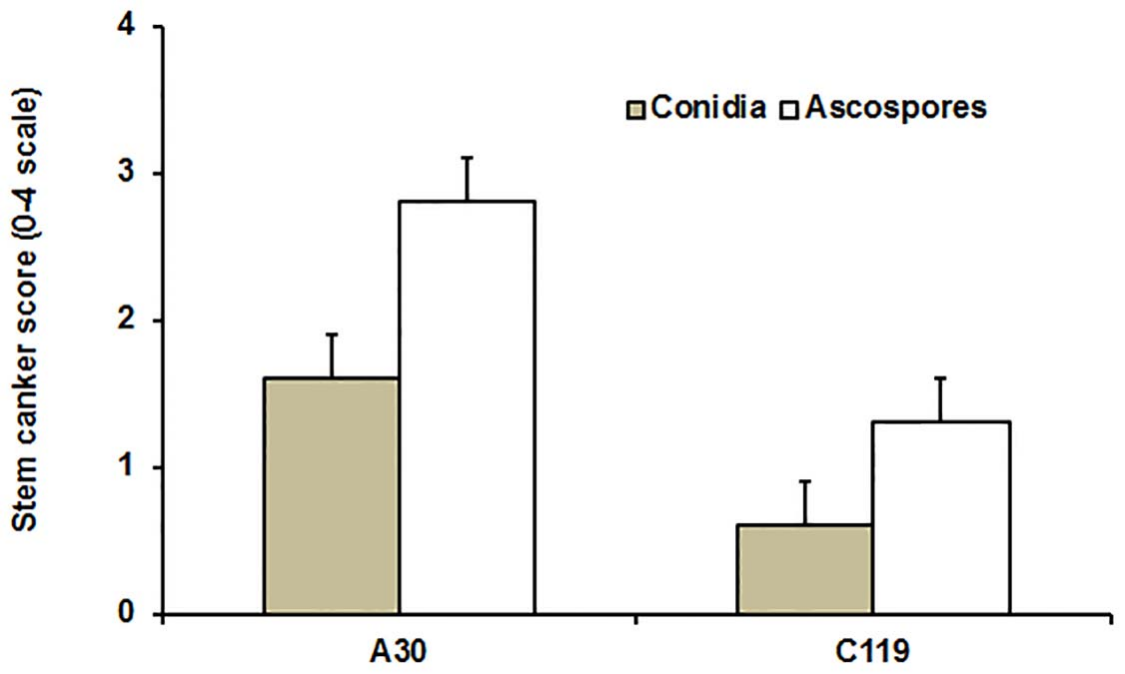
Severity of phoma stem canker symptoms. Leaf petioles of oilseed rape doubled haploid lines A30 (susceptible) or C119 (with quantitative resistance) were inoculated with conidia of isolate ME24/3.13 or ascospores of *Leptosphaeria maculans*; severity of stem canker was assessed on a 0–4 scale at 46 days post inoculation (dpi) (ascospore inoculum) or 87 dpi (conidial inoculum). Bars show standard errors (17 d.f. for conidial inoculation; 50 d.f. for ascospore inoculation).

**Figure 8:**
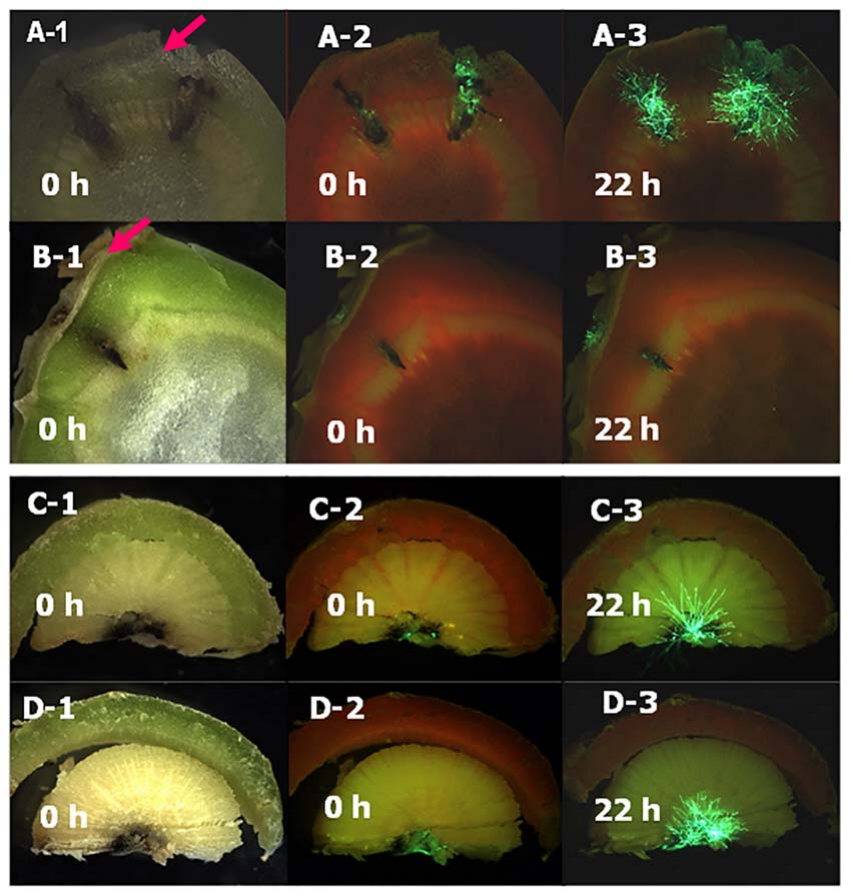
Growth of *Leptosphaeria maculans* in the stem cortex or pith. Leaf petioles of oilseed rape doubled haploid lines A30 (susceptible) or C119 (with quantitative resistance) were inoculated with conidia of GFP-expressing *L. maculans* isolate ME24/3.13, stems of A30 (A, C) and C119 (B, D) were cut horizontally at the leaf scar (A, B) of the inoculated leaf or at the hypocotyl (C, D) 2–3 cm below the leaf scar at 87 days post inoculation (dpi). Stem cross-sections were viewed with brightfield illumination (A-1, B-1, C-1, D-1) or a GFP2 filter (A-2-3, B-2-3, C-2-3, D-2-3) immediately after cutting (0 h) or after incubation for 22 hours in darkness at 20°C (22 h).

For leaf petioles inoculated with conidia of GFP-expressing isolate ME24/3.13, although no visible symptoms were observed at the leaf scars (Fig. 6A-1) at the time when the inoculated leaves abscised, growth of *L. maculans* in the leaf scar tissues was observed by using a GFP filter (Fig 6A-2) at 25 dpi. By 36 dpi, visible stem canker symptoms had started to develop at the leaf scars on some plants of line A30 (Fig 6B-1), as confirmed by observing GFP fluorescence (Fig 6B-2). Necrotic stem canker lesions were assessed at 87 dpi; there was a significant difference (*P*<0.05, 17 d.f., SED 0.96) between A30 and C119 (Fig. 7), with stem canker more severe on A30 (Fig. 6C) than on C119 (Fig. 6D). After it reached the stem, GFP-expressing *L. maculans* was observed to spread both vertically up/down the stem and horizontally towards the stem pith. GFP-expressing *L. maculans* was observed in the pith of more A30 plants (80%) than C119 plants (56%) and spread further both up and down internal stem tissues of A30 (27.7 mm) than those of C119 (14.8 mm). After incubation of the stem cross-sections in darkness for 22 h, *L. maculans* grew more slowly in the cortex of C119 (Fig. 8B) than in that of A30 (Fig. 8A) but there was no difference between C119 (Fig. 8 D) and A30 (Fig. 8 C) in *L. maculans* growth after *L. maculans* had reached the stem pith.

There were differences between conidial inoculum and ascospore inoculum in incubation period for phoma stem canker development, with the incubation period shorter for ascospores than for conidia. Phoma stem cankers were observed at 25 dpi for plants inoculated with ascospores and at 36 dpi for plants inoculated with conidia. Stem canker developed more rapidly and more severely on plants inoculated with ascospores than on plants inoculated with conidia (Fig. 6C-F; Fig. 7).

In experiments with ascospore inoculum (PExpt 2, 3 & 4), 42% of inoculated leaves of A30 and 38% of those of C119 were senescent by 25 dpi. Stem canker developed more rapidly in A30 than in C119. By 32 dpi, 63% of inoculated leaves of A30 and 8% of inoculated leaves of C119 had developed stem canker symptoms at their leaf scars in PExpt 3; 60% of inoculated leaves of A30 and 30% of inoculated leaves of C119 had developed stem canker symptoms at their leaf scars by 33 dpi in PExpt 4. By 46 dpi, the internal stem canker severity score (average of PExpt 2, 3 & 4) was significantly greater in A30 than in C119 (*P*<0.001, 50 d.f., SED 0.27) (Fig. 7); the amount of *L. maculans* DNA was significantly greater in stems of A30 than in stems of C119 (*P*<0.05, 50 d.f., SED 1.48; Fig. 4B). However, there was a significant difference between the three experiments in stem canker severity (*P*<0.01, 50 d.f., SED 0.28) and in the amount of *L. maculans* DNA in stem tissues (*P*<0.05, 50 d.f., SED 1.59). There was no significant interaction between experiment and DH line in either stem canker severity or the amount of *L. maculans* DNA.

There were significant linear relationships between amount of *L. maculans* DNA (*n*) in stem tissues and stem canker severity score (*s*) in experiments PExpt 2 (*n* = 0.87*s*–1.61, *R* ^2^ = 0.86, *P*<0.001, 7 d.f.) and PExpt 3 (*s*) (*n* = 0.24*s*+2.70, *R* ^2^ = 0.56, *P*<0.001, 21 d.f.) (Fig. 5D). Differences between these two regression lines in both the slope (*P*<0.001) and the intercept (*P*<0.001) were significant. There was no significant linear relationship between amount of *L. maculans* DNA in stem tissues and stem canker severity score in experiment PExpt 4.

## Discussion

Results of these experiments with DH lines C119 and A30 suggest that *B. napus* quantitative resistance against *L. maculans* can be detected in young plants in controlled conditions by leaf lamina or leaf petiole inoculation. These methods should be further evaluated on a wide range of cultivars or breeding lines with different levels of quantitative resistance to develop methods can be used reliably by breeders to select for quantitative resistance in young plants. This will not only save money but also accelerate the process of breeding for resistance. Traditionally, breeding for quantitative resistance against *L. maculans* in winter oilseed rape has relied on disease assessments before harvest 10–11 months after sowing of field experiments [3], [18], [30]. By contrast, an assessment of quantitative resistance in young plants in controlled conditions takes only 1–2 months by leaf lamina inoculation or 2–3 months by leaf petiole inoculation (Table 2). Furthermore, controlled environment assessment of young plants can test large numbers of lines. Such controlled environment assessment methods could be used for pre-selection of breeding material to decrease the number of lines required for final field testing and could thus save costs.

**Table 2:** Comparison of different methods for assessing *Brassica napus* quantitative resistance against *Leptosphaeria maculans*.

Inoculum	Assessment method	Advantages	Disadvantages	Duration	Ref. 1
Field expt., natural inoculation					
Ascospores	Score canker severity on stem before harvest	Test of field resistance under natural conditions, reliable results, no need to wound leaf	Variation between years and regions, costly to assess large numbers of lines, cannot distinguish quantitative from *R* gene resistance when effective *R* gene is present in the plant	10–11 months^2^	1, 2, 3, 4
CE expt., cotyledon inoculation – growth in hypocotyl					
Conidia	Score canker severity on hypocotyl	Easy to produce genetically homogeneous inoculum, can test large numbers of plants	Tests few isolates, less infective than ascospores, variation between expts, not easy to produce canker symptoms, hypocotyl infection does not occur in field in Europe	60–90 days	5
CE expt., leaf lamina inoculation – growth in leaf petiole					
Conidia	Extent (distance) of visible necrosis or GFP	Easy to produce homogeneous inoculum, rapid, can test large numbers of plants, visualize symptomless growth if GFP used	Tests few isolates, less infective than ascospores, need to wound leaf, may not distinguish lines with moderate resistance, restriction on use of GFP	40–45 days	3, 6
Ascospores	Extent (distance) of visible necrosis, qPCR	Easy to assess, tests natural population, can test large number of plants, no need to wound leaf, can distinguish quantitative from *R* gene resistance	Difficult to produce large amount of inoculum, inoculum not genetically homogeneous, may not distinguish moderate resistance lines	35–40 days	6
CE expt., leaf petiole inoculation – growth in stem tissue					
Conidia	Extent of visible necrosis or GFP	Easy to produce genetically homogeneous inoculum, visualize symptomless growth if GFP used	Tests few isolates, less infective than ascospores, restriction on use of GFP	70–90 days	6
Ascospores	Extent of visible necrosis, qPCR	Easy to assess, test natural population, results close to natural conditions, reliable results	Difficult to produce large amount of inoculum, inoculum not genetically homogeneous, cannot distinguish quantitative from *R* gene resistance when effective *R* gene is present	50–60 days	6

1 1,Delourme et al., 2006; 2, Fitt et al., 2006; 3, Huang et al., 2009; 4, Pilet et al., 1998; 5, Travadon et al., 2009; 6, this study.

2 To obtain a reliable estimate, it is necessary to do several experiments at different sites in different seasons due to G × E interactions.

Differences between A30 and C119 in growth of *L. maculans* in leaf petioles and stems suggest that quantitative resistance against *L. maculans* decreases the severity of stem canker by impeding the growth of *L. maculans* in both petiole and stem tissues. The shorter distance grown by *L. maculans* in petioles of C119 than in those of A30 suggests that quantitative resistance in C119 impeded the growth of *L. maculans* along the petiole towards the stem. However, in previous work with commercial cultivars Darmor (with quantitative resistance) and Eurol (without quantitative resistance), there was no difference between the two cultivars in growth of *L. maculans* along the petiole although there was a difference between them in severity of stem canker [6]. The observation that growth of GFP-labelled *L. maculans* was slower in the stem cortex of C119 than in the cortex of A30 but did not differ between A30 and C119 after *L. maculans* reached the stem pith suggests that quantitative resistance in C119 mainly operated during growth of *L. maculans* from the stem cortex to the stem pith. This is consistent with results of previous work [6]. The development of more severe necrosis in hypocotyls of A30 than in those of C119 [28] suggests that quantitative resistance against *L. maculans* may also impede the growth of the pathogen from the cotyledon to the hypocotyl. There is a need to test a wide range of cultivars and DH lines under different conditions to investigate the relationship between quantitative resistance against *L. maculans* in leaf petiole and stem tissues identified in controlled environment experiments and quantitative ‘field’ resistance identified by end of cropping season disease assessments in field conditions.

Results of these experiments with C119 and A30 inoculated with either conidia or ascospores suggest that leaf lesion area might be used to assess quantitative resistance against *L. maculans* at stage 1 (Fig. 1). The good positive relationship between leaf lesion area and distance grown by *L. maculans* along the petiole towards the stem suggests that *L. maculans* may reach the stem more quickly and subsequently cause more severe stem canker before harvest if it is growing from large leaf lesions than if it is growing from small leaf lesions. This suggests that the measurement of areas of phoma leaf lesions might provide a more accurate assessment of quantitative ‘field’ resistance than assessing the numbers of phoma leaf lesions in field experiments in autumn since there is a poor relationship between the number of phoma leaf lesions in autumn and the subsequent severity of stem canker in summer [38]. Furthermore, it is quicker and more reliable to measure lesion area than to measure distance grown by *L. maculans* along leaf vein/petiole. For example, with conidial inoculation (e.g. LExpt 1 &2), lesion area can be measured at 17–18 dpi and no difference was observed between experiments. However, distance grown along leaf petiole cannot be measured until 22–25 dpi and differences between experiments were observed. Measurement of leaf lesion area is easy and simple. If area of leaf lesions could be used to assess quantitative resistance in field conditions, it would be a valuable method for breeders. Leaf lesion area has been used to assess quantitative resistance against other fungal pathogens [5], [39]–[40]. However, there is a need to test a wide range of cultivars under different conditions to investigate the relationship between the area of phoma leaf lesions in autumn and the severity of phoma stem canker in the following summer.

Comparison of different inoculation methods for assessing *B. napus* quantitative resistance against *L. maculans* in young plants suggests that leaf petiole inoculation is better than other methods for use in controlled environments (Table 2). Although canker symptoms can be produced on hypocotyls after cotyledon inoculation [28], production of canker symptoms requires 4 weeks at 6°C followed by 6 weeks at 20°C and hypocotyl infection is not representative of what happens in field conditions in the UK. Furthermore, the structure of hypocotyl is different from that of stem [41]. Although the leaf lamina inoculation method is quicker than leaf petiole inoculation, the main damage caused by *L. maculans* is canker on stems, and results of leaf lamina inoculation experiments need to be further confirmed by assessing canker severity on stems. The consistency of the results of the four petiole inoculation experiments for stem canker score (i.e. less severe stem canker on C119 than on A30) suggests that quantitative resistance against *L. maculans* can be reliably assessed in young plants during colonisation of stem tissues (stage 2) after petiole inoculation. This conclusion is supported by previous work on cultivars Darmor and Eurol [6].

Results of these experiments with different types of inoculum suggest that ascospore inoculum of *L. maculans* is more effective than conidial inoculum in terms of leaf lesion and stem canker development. The incubation periods for leaf lesion and stem canker development may have been shorter for ascospore inoculum than for conidial inoculum because ascospores germinate more rapidly and contain more nutrients for the pathogen to colonise the host than conidia. Although phoma stem canker epidemics are initiated by ascospores released from crop debris, much experimental work with young plants to study resistance has used conidial inoculum [8], [33], [42]. Use of ascospores from natural populations as inoculum can simulate what happens in natural conditions and is more likely to identify ‘quantitative resistance’ expressed in field conditions. Recent work has shown that some components of quantitative resistance against *L. maculans* can be isolate-specific [43]; use of conidia of single *L. maculans* isolates may not accurately assess quantitative resistance while use of ascospore inoculum from natural populations is more likely to do so. Therefore, use of ascospore inoculum is more reliable than use of conidial inoculum for detecting and assessing quantitative resistance.

Comparison of different assessment methods used in controlled environments suggests that leaf lesion area could be a good measurement for use at stage 1 and stem canker severity score could be a good measurement for use at stage 2 (Table 2). At stage 1, measurement of lesion area was better than the other three methods used (GFP, qPCR and visible distance grown) and it might be used in field experiments whereas it is difficult to measure the spread of *L. maculans* from the leaf lesion along the leaf petiole in field conditions. In addition, assessment of leaf lesions (appearance and size) can distinguish between quantitative resistance (typical large grey lesion) and *R* gene-mediated resistance (small dark lesion) against *L. maculans* [6], [9]. Although GFP and qPCR are valuable methods for assessing symptomless growth of *L. maculans* in leaf or stem tissues, GFP can be used only in controlled environments and is not practical for use with large numbers of lines. Whilst qPCR can be used with large numbers of lines, it is expensive and not always reliable. Because qPCR is very sensitive and *L. maculans* has a symptomless growth phase, *L. maculans* DNA was detected in stems (Fig. 5D) or hypocotyls [28] with no visible necrosis. By contrast, scoring stem canker severity in controlled environment experiments is more reliable at stage 2. More severe stem cankers developed on A30 than on C119 in all the four experiments with petiole inoculation. Results of stem canker assessment in young plants in controlled conditions (stage 2) could be used to predict how the lines or cultivars will operate in field conditions.

Development of methods to phenotype quantitative resistance against plant pathogens in young plants under controlled conditions will improve the process of breeding cultivars for control of crop diseases. Assessment of quantitative trait loci (QTL) for resistance is often inconsistent due to interactions with environment [4], [16], [31]. Detection of quantitative resistance in controlled conditions can avoid the influence of fluctuations in natural weather conditions. Furthermore, with the development of methods to assess quantitative resistance in controlled environments, it is possible to test effectiveness of the quantitative resistance under a range of different controlled conditions, which will help to optimise the use of quantitative resistance in different natural environments. Due to the incomplete nature of quantitative resistance and the difficulties in assessing it, mechanisms of operation of quantitative resistance are poorly understood [4]. Quantitative resistance has been thought to be expressed at later stages of plant development and it has been referred to as ‘adult plant resistance’. There has been little work to investigate potential operation of quantitative resistance in young plants; results of this work suggest that quantitative resistance can be expressed at early stages of plant development. With the use of next generation sequencing techniques and reduced costs, more genome sequences of both pathogens and their hosts are available [44]–[47]. Identification of stable QTL in controlled conditions will improve our understanding of mechanisms of operation of quantitative resistance, which will help in the breeding of cultivars with durable resistance to contribute to food security.
